# Preeclampsia prevention: a case-control study nested in a cohort

**Published:** 2015-12-30

**Authors:** Alberto Alzate, Rodolfo Herrera-Medina, Lucia M Pineda

**Affiliations:** 1 Grupo de Investigación en Epidemiología y Servicios (GRIEPI). Universidad Libre-seccional Cali, Colombia; 2 Coomeva EPS. Cali, Colombia

**Keywords:** Preeclampsia, calcium, conjugated linoleic acid, prevention, adolescence

## Abstract

**Background:**

Preeclampsia is the main complication of pregnancy in developing countries. Calcium starting at 14 weeks of pregnancy is indicated to prevent the disease. Recent advances in prevention of preeclampsia endorse the addition of conjugated linoleic acid.

**Objective:**

To estimate the protective effect from calcium alone, compared to calcium plus conjugated linoleic acid in nulliparous women at risk of preeclampsia.

**Methods:**

A case-control design nested in the cohort of nulliparous women attending antenatal care from 2010 to 2014. The clinical histories of 387 cases of preeclampsia were compared with 1,054 normotensive controls. The exposure was prescriptions for calcium alone, the first period, or calcium plus conjugated linoleic acid, the second period, from 12 to 16 weeks of gestational age to labor. Confounding variables were controlled, allowing only nulliparous women into the study and stratifying by age, education and ethnic group.

**Results:**

The average age was 26.4 yrs old (range= 13-45), 85% from mixed ethnic backgrounds and with high school education. There were no differences between women who received calcium carbonate and those who did not (OR= 0.96; 95% CI= 0.73-1.27). The group of adolescents (13 to 18 years old) in the calcium plus conjugated linoleic acid was protected for preeclampsia (OR= 0.00; 95% CI= 0.00-0.44) independent of the confounder variables.

**Conclusions:**

1. Calcium supplementation during pregnancy did not have preventive effects on preeclampsia. 2. Calcium plus Conjugated Linoleic acid provided to adolescents was observed to have preventive effect on Preeclampsia.

## Abstract (es)

**Introducción:**

La preeclampsia es la principal causa de mortalidad materna y bajo peso al nacer en países en vías de desarrollo. Empezar la suplementación de calcio en la semana 14 de gestación es indicada para prevenir la enfermedad. Recientes avances en prevención de preeclampsia han sugerido la suplementación de calcio y la combinación de calcio con ácido linoleico conjugado.

**Objetivo:**

Estimar el efecto protector de la suplementación de calcio (CC), comparado con la suplementación de calcio más ácido linoleico conjugado (CC+ALC) en mujeres embarazadas primigravidas con relación al desarrollo de preeclampsia.

**Metodos:**

Se realizó un estudio de casos y controles anidado en una cohorte retrospectiva de mujeres embarazadas primigravidas que asistieron al programa de control prenatal entre el año 2010 hasta el años 2014. La historia clínica de 387 casos de preeclampsia fueron comparadas con 1,054 controles en pacientes embarazadas primigestantes, normotensas. Las gestantes expuestas fueron consideradas como aquellas gestantes que recibieron solo calcio, en el primer periodo de evaluación, o calcio más ácido linoleico conjugado, segundo periodo de evaluación, desde las semanas 12 a la 16 del embarazo. Los posibles factores de confusión se controlaron admitiendo solo las primigestantes y estratificando, por edad, educación y grupo étnico de la paciente.

**Resultados:**

La edad promedio de las pacientes fue de 26.4 años (rango 13-45), 85% con etnia mestiza y educación secundaria completa. La exposición a CC+ALC tuvo un efecto protector en las adolescentes (13 a 18 años) (OR= 0.00, IC 95%= 0.00-0.44; *p*= 0.005), mientras que la exposición a CC no modificó la incidencia de preeclampsia (OR= 0.96, IC 95% %= 0.73-1.27; *p*= 0.82).

**Conclusión:**

1. La suplementación de solo calcio durante el embarazo no tuvo efecto preventivo para la preeclampsia, 2. La suplementación con calcio y ácido linoleico conjugado previno la aparición de preeclampsia en adolescentes.

## Introduction

Preeclampsia, principal cause of maternal and perinatal mortality, as well as restriction in intrauterine growth and low birth weight in developing countries, is a multifactorial disease of unknown cause for which diverse studies have been conducted with preventive purposes 1. Calcium supplementation during pregnancy revealed some promising results for the prevention of this disease 2; however, later studies carried out in various countries with population samples observed that calcium does not prevent preeclampsia 3. It was demonstrated that calcium reduces by one third the presentation of eclampsia and has effects on reducing the risk of perinatal mortality in adolescents 4, which is why it has been recommended as a nutritional measure during pregnancy, bearing in mind that in developing countries a deficit exists in nutrient intake during pregnancy, especially in adolescent population 5. Calcium reduces parathormone levels during pregnancy 6, but does not reduce concentrations of intracellular calcium, does not improve endothelial function, and does not improve production of vasodilatory prostaglandins; all the aforementioned is indeed produced by the combination with conjugated linoleic acid, factors recognized as protective for preeclampsia development 7-9.

The population effect of calcium supplementation and linoleic acid to reduce the risk of preeclampsia was initially demonstrated in an open study, which administered 1,443 treatments to low-income pregnant women from the western part of Colombia, observing a significant reduction in preeclampsia incidence 10.

This study sought to compare the effects of administering calcium carbonate with the administration of calcium citrate plus conjugated linoleic acid for preeclampsia prevention in primigravidae patients.

## Materials and Methods 

**Figure 1.**
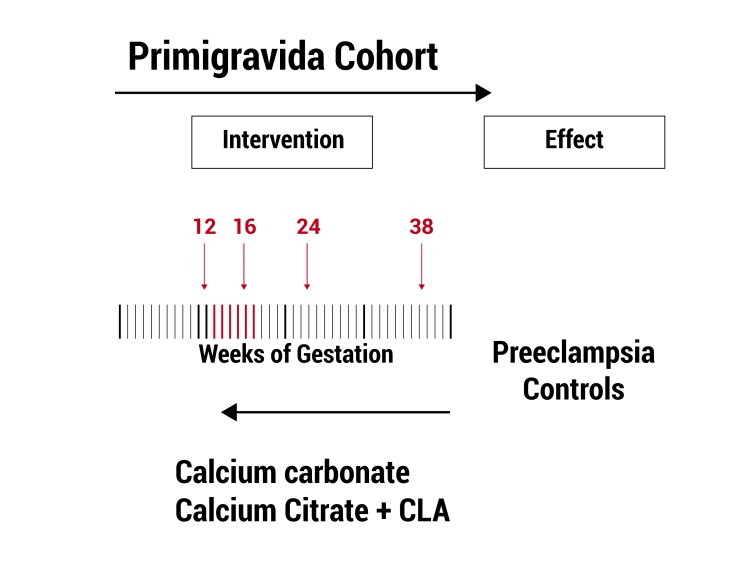
** Figure 1.** Evaluation of the intervention: from left to right the cohort of pregnant women advances; from right to left the evaluation ask if the effect-preeclampsia- is related or not to the intervention CC or CC+CLA, since week 12-16 of the pregnancy.

A case-control study was conducted nested in a cohort of primigravidae women in Cali, Colombia, in a healthcare promoter company, who received calcium carbonate (CC) or calcium plus conjugated linoleic acid (CC+CLA) combination since weeks 12-16 of the pregnancy (Fig. 1). 

### Inclusion criteria

Pregnant patients in primiparous and normotensive state (PA <140/90 mm Hg) and adherence to prenatal control (at least three prenatal controls) without basic obstetric pathologies. Voluntarily accepted to enter the study before week 12 of gestation. 

###  Exclusion criteria

Patients who did not comply with the inclusion criteria; when the information contained in the clinical history was insufficient and, when there was no prescription evidence or such evidence had not been claimed in the pharmacy.

### Selection criteria 

**Figure 2.**
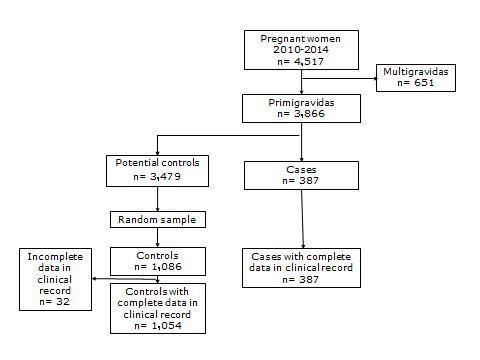
Selection criteria of cases and controls for study with exposure to calcium carbonate or calcium plus conjugated linoleic acid, Cali-Colombia 2010-2014.

Development or not of preeclampsia (arterial hypertension induced by the pregnancy accompanied by proteinuria >300 mg/24 h urine) and exposed or not to CC or CC+CLA (Fig. 2).

### Methodology

The study was based on the organization's electronically systematized clinical and administrative records. 

The evaluation was conducted over two well-defined periods. During the first period, from 01 January 2010 to 31 December 2012, only calcium carbonate (600 mg/day/elemental calcium) was used. During the second period from 01 January 2013 to 31 January 2014, the pregnant women began to receive CC+CLA (600 mg/day/elemental calcium and 450 mg/day of conjugated linoleic acid). 

Cases were defined as patients with diagnosis of preeclampsia (pregnancy-induced hypertension (≥140/90 mm Hg of blood pressure with significant proteinuria (≥300 mg/24 h). Controls were defined as patients with term pregnancy through vaginal or cesarean delivery, without pathology or with pathology different from the case, with adherence to prenatal control. For each case identified during each month, three controls were chosen through random sampling.

Exposure to CC or CC+CLA was obtained from two sources. A search was made in the clinical histories of all the records corresponding to medical and nutritional prescriptions, looking for dates in which such drugs were prescribed and the corresponding week of the pregnancy. Secondly, in the drug-surveillance database, through the affiliates code, an equal search was conducted, comparing the results. For CC+CLA, the study kept records, at the start of the exposure, the date on which the pharmacy delivered the medication to the pregnant woman.

During the evaluation of a preventive measure like the administration of calcium during weeks 12 to 16 of the pregnancy, it is expected that the reduction in the number of cases of the disease would be at least 50% (if the measure is effective). With these assumptions, the sample size was calculated with 95% confidence level (α= 0.05), a power (1-β) of 80%, and exposure to CC or CC+CLA in 80% of the controls, 186 cases and 186 controls were calculated (EpiInfo^®^ version 6.04. Centers for Disease Control and Prevention, CDC. Atlanta GA, USA, 2001). With 3 controls per case, 120 cases and 360 controls were needed. When stratification was introduced in the analysis with three groups, the prior sample size was increased threefold to reach 360 cases and 1,080 controls.

Univariate and bivariate analyses were performed using Chi^2^, Fisher's exact probability test, and Odds Ratios with their respective confidence intervals of 95%. The stratification during the analysis was used to guarantee the homogeneity of the data: per age group (13-18, 19-35, and 35-45 yrs). The study used the estimator of the global OR and Chi^2^ by Mantel & Haenszel. In the multivariate analysis, the complete model was estimated for the logistic regression, including preeclampsia as response variable and as explanatory to all the variables resulting in the bivariate analysis with statistically significant values, in addition to age independent of its statistical value. The model's performance and goodness of fit were evaluated through the analysis of residuals (STATA^®^, version 6). The study was approved and monitored by the Organization's institutional ethics committee for research studies on humans.

## Results

For the period between 2010 and 2014, a total of 4,517 records of deliveries were found of which 3,866 corresponded to primigravidae patients. Finally, 387 were selected corresponding to the case diagnosis (Table 1) and, through random sampling the 1,086 controls were selected with diagnosis of normal delivery. After reviewing the clinical histories, 32 histories were discarded due to incomplete data or transfers to other cities, for a total of 1,054 controls (Fig. 2). The mean age of the patients studied was 26.4 yrs (range= 13-45), 85% of mixed ethnic origin with complete high school education.

**Table 1.** Preeclampsia cases in 1,441 recruited pregnant women studied Cali-Colombia 2010-2014.

ICD-10	Diagnosis	Number	%
014.00	Moderate Preeclampsia	11	2.8
014.01	Severe preeclampsia	88	22.7
014.9	Unspecified preeclampsia	261	67.4
014.90	Preeclampsia during pregnancy	23	5.9
014.93	Preeclampsia during Delivery	1	0.3
014.93	Preeclampsia during puerperium	3	0.8
	Total	387	100.0

The cases and controls selected showed a similar structure regarding education (Table 2); education in these patients was high, with 28.0% of the women being professionals. A difference was found in the participation of Afro-American women, 15.0% in cases and 8.6% among the controls (OR= 1.68, 95% CI= 1.14-2.48).

**Table 2.** ** Table 2. **Sociodemographic characteristics of preeclampsia cases and normotensive controls (N= 1,441), Cali-Colombia 2010-2014.

Variable	Cases		Controls		total		*p* value
	Number	%	Number	%	Number	%	
Age (yrs)							
13-18	49	12.7	179	17.0	228	15.8	
19-34	301	77.8	827	78.5	1,128	78.3	
35-45	37	9.6	48	4.6	85	5.9	
Total	387	100.0	1,054	100.0	1,441	100.0	0.0004
Education							
None	0	0.0	1	0.1	1	0.1	
Incomplete primary	4	1.0	7	0.7	11	0.8	
Complete primary	4	1.0	11	1.1	15	1.1	
Incomplete high school	49	12.8	125	12.1	174	12.3	
Complete high school	111	28.9	353	34.1	464	32.7	
Technical	65	16.9	166	16.0	231	16.3	
Technological	41	10.7	84	8.1	125	8.8	
Professional	110	28.6	287	27.8	397	28.0	
Total	384	100.0	1,034	100.0	1,418	100.0	0.4387
Ethnic group							
Afro-American	58	15.0	91	8.6	149	10.3	
The rest	328	85.0	960	91.4	1,288	89.7	
Total	386	100.0	1051	100.0	1,437	100.0	0.0004

Start of prenatal control in 52.9% of the cases was prior to week 10; start of the calcium prescription in 32.3% of the cases was prior to week 16. Administration of CC+CLA before week 16 was similar for cases and controls (Table 3). No association was found between the prescription of only calcium before week 16 and the diagnosis of preeclampsia (adjusted OR= 0.96, 95%CI= 0.73-1.27); the same was found for CC+CLA administered before week 16 (adjusted OR= 1.0, 95% CI= 0.70-1.42); however, when stratifying by age it was evidenced that primigravidae patients under 18 years of age were protected (OR= 0.00, IC 95%: 0.00-0.44) because none of the adolescents receiving CC+CLA before week 16 manifested the disease. This finding is statistically significant (Chi^2^= 7.69, *p*= 0.0055). In the other age groups no statistically significant association was found between the disease and the protective measure (Table 3).

**Table 3:** ** Table 3.** Adjusted association between nutritional supplements by preeclampsia and normotensive controls by age groups (N=1,441), Cali-Colombia, 2010-2014.

Age (yrs)	Treatment		Preeclampsia	Controls	OR	95% CI	p value
13-18	CC	Yes	13	40	1		
		No	28	115	1.33	0.59-3.00	0.5763
	CC+CLA	Yes	0	29	1		
		No	49	150	0.00	0.00-0.44	0.0055
19-34	CC	Yes	82	244	1		
		No	174	489	0.94	0.69-1.30	0.7710
	CC+CLA	Yes	57	131	1		
		No	244	696	1.24	0.86-1.78	0.2526
35-45	CC	Yes	11	26	1		
		No	20	20	0.42	0.15-1.20	0.1100
	CC+CLA	Yes	4	3	1		
		No	33	45	1.82	0.31-11.30	0.1100

CC: Calcium prescribed before 16 weeks of gestational age

CC+CLA: Calcium-conjugated linoleic acid before 16 weeks of gestational age

In the multivariate analysis, when comparing the crude and adjusted OR by age of patient, ethnicity, and educational level, both in patients with calcium carbonate as in those with CC+CLA the results were similar and indicate that these variables, associated to risk of preeclampsia, are not acting as confounders in the study. The risk for Afro-American pregnant women was high (crude OR= 1.86 and adjusted OR= 1.68) and statistically significant (95% CI= 1.14-2.48). Higher education did not show protective effect (adjusted OR= 0.76, 95% CI= 0.56-1.04).

## Discussion

According to the 2014 report by the United Nations Development Program (UNDP-Colombia), 99% of maternal mortality occurs in developing countries, mainly affecting vulnerable populations from rural areas and low-income patients. In Colombia, the group of pregnant adolescents was the hardest hit with this condition, which gives relevance to the results of this study. 

The sample size (387 cases and 1,054 controls) was enough to bear internal validity and allow reaching conclusions. Ethnic factors were associated to preeclampsia, as observed in previous studies 11; however, this was not the preliminary objective of the study. Calcium supplementation during pregnancy did not have preventive effects on preeclampsia and said effects in adolescents were observed with the addition of conjugated linoleic acid. These results have biologic plausibility and external validity 2-4,7-10.

Although only calcium is routinely used during prenatal control in many countries, given its undoubted utility as nutritional intervention, its use alone to prevent preeclampsia has had contradictory results since 2001 3,4, 12. The pregnant women included in this study were the representative number sufficient to detect differences in a pregnant population with high educational level and adequate income, given that it is a population ensured within the Colombian Social Security System, a group in which the opportunity is guaranteed in medical care and its quality. Due to the types of patients included in the study, nutritional limitations should not exist in calcium intake in their normal diet. Prenatal control was early in most patients, without significant difference in supplying supplements among the groups related to gestational age (Table 3), controlling this potentially confounding factor; this study did not show a preventive effect on preeclampsia with the use of only calcium and the effect of the ethnicity as a risk factor agreed with the results from previous studies 3,5,11. The only difference between calcium from a salt like carbonate and another salt like citrate tetrahydrate is the carbon dioxide (CO_2_) released in the first and the water in the latter because what is absorbed in the blood is the free ion and it acts functionally. 

Over a decade ago, publications appeared demonstrating the complementarity of the results obtained through observational studies and those from randomly controlled clinical trials 13. The need to evaluate the effect of treatments resulting from clinical trials in well-selected populations, compared to the same treatments in populations with patients quite different from each other, leads to the creation of the initiative known as Good Research for Comparative Effectiveness (GRACE) 14, to "provide evidence that fills the voids that remain on the behavior of subgroups of especial interest, broader populations, and results of the medication under normal conditions of their application in healthcare services". The International Society for Pharmacoeconomics and Outcomes Research (ISPOR) recommends guaranteeing the internal validity and the capacity for causal inference from observational studies on retrospective databases 15, given that the lack of randomness generates difficult-to-control biases and confounders (mix of effects). The ISPOR guide 16,17 indicates how to address this problematic from the design and analysis of the results, by following the same guidelines from other initiatives like Strengthening the Reporting of Observational Studies in Epidemiology (STROBE) 18.

The cases-and-controls methodology applied by following the guidelines from the GRACE proposal 14, showed its effectiveness and the comparability of the initial findings from the clinical trial, with the evaluation made in this study. The quality of the records and ease in finding clinical histories stored electronically permitted using this type of evaluation on records, which opens new evaluation possibilities.

Selection biases were controlled by this being a nested study of cases and controls on a cohort of pregnant patients, taking the women who began prenatal control before week 12, both in cases as in controls, guaranteeing their having the same probability of being exposed to the protective measure (calcium during weeks 12 to 16) and without still manifesting the disease. The detection bias of the case or non-case was minimized with the of primigravidae women who received calcium during this period. Classification bias was controlled by detecting and discarding clinical histories of poor quality.

Possible confounding factors were controlled by stratifying, on the one hand, in the analysis of the patient's age variable, and on the other, by controlling the number of prior pregnancies, admitting only primigravidae participants.

The following remained beyond the reach of the study: genetic factors, cigarette smoking, alcohol intake, and other events like undeclared prior abortions, which do not intervene between exposure and the result. Between the exposure and the result the only factor that mediates and which we ignore is the number of doses taken by the patient, and it is uncontrollable, but it is equally foreseeable to occur in equal manner between cases and controls.

The lack of protective effect from calcium alone on preeclampsia was demonstrated preliminarily in studies conducted in North America (patients with high socioeconomic status who received supplementation of 2 g/day of elemental calcium) (N= 4,589) 3, similar to the socioeconomic status of the pregnant women in the present study; thereafter, the same negative result was observed in another study carried out in various countries (N= 8,325) (supplementation of 1.5 g/day of elemental calcium) pregnant participants with low basal intake of calcium in their diet, with the assumption that it was a confounder 4.

A recent meta-analysis evaluated nine controlled clinical trials with supplementation of low doses of calcium (<1 g/day/elemental calcium) with relation to preeclampsia development, under the premise that the supplementation of high doses from the first studies (1.5-2.0 g/day of elemental calcium) 3,4 has had logistics problems, especially in low-income countries, and has observed low tolerance and lack of adhesion to the protocols; study that noted a protective effect 19 (N= 2,234, RR= 0.38, 95% CI= 0.28-0.52), coherent with the low doses of calcium used in the supplementation of CC+CLA in the present study (600 mg/day of elemental calcium).

In regions with low basal intake of conjugated linoleic acid in their diet (Germany: 350 mg/day, north Finland: 90 mg/day, south Finland: 310 mg/day) preeclampsia incidence was high (15.9, 13.9, and 7.9%, respectively) 20,21; in contrast, in regions with a high basal intake of conjugated linoleic acid in their diet (the United States 1,000 mg/day, Australia 1,800 mg/day) preeclampsia incidence was low (5.0-4.2%) 22. Conjugated linoleic acid is a combination of isomers from the intake of linoleic acid, produced at intestinal level and in the mammary gland, with humans having levels in minimum amounts with physiological effects 23-25. It is known that biochemical changes during preeclampsia are similar to those of metabolic syndrome (arterial hypertension, hyperlipidemia, low HDL, and insulin resistance). It has been observed that with the supplementation in animals with conjugated linoleic acid there is reduced inflammation, hyperlipidemia, and insulin resistance, known risk factors for preeclampsia development 26,27; interestingly, conjugated linoleic acid is, on its own, capable of reverting the metabolic syndrome in humans 28; previous studies have demonstrated that the combination with calcium is needed to induce protective mechanisms for preeclampsia 7-9. For this study, the association of preeclampsia and Afro-American ethnicity was a significant risk factor, which has also been previously described 11; however, it must be considered that this study was originally not designed to evaluate this association.

Among the limitations of this study is that it was conducted in a population insured by the General System of Social Security with high educational level, similar to the population from developed countries and not to the general population of Colombia; more than half the patients received early calcium intervention, which is not common in the general population; however, it is a strength to assess the effect of interventions in the present study.

The results obtained with CC+CLA were convincing, given the total protection found in the group younger than 18 yrs of age. The CC+CLA intervention reduces intracellular calcium, which reduces concentrations of cyclic GMP and can induce vasodilation 7-9. Previous studies with only calcium did not modify the intracellular calcium levels 29 it is of specific interest that one of the protective effects observed in the international multicentric study was with adolescents 5,7-9.

This finding demonstrates that in the most vulnerable group, that of adolescents, supplementing with CC+CLA is protective, as found in controlled clinical trials 7-9,30. The evaluation compared CC and CC+CLA, which had not been done previously. These results show the advantage of the addition of conjugated linoleic acid in primigravidae adolescents. 

## Conclusion

Supplementation with CC+CLA prevented the onset of preeclampsia in adolescents, which was not observed with the supplementation of CC alone.
